# Focal screening and treatment for malaria: defining the sentinel population

**DOI:** 10.1186/1475-2875-13-S1-P86

**Published:** 2014-09-22

**Authors:** Gillian H Stresman, Amrish Baidjoe, Jennifer Stevenson, Wycliffe Odongo, Chrispin Owaga, Elizabeth Marube, Victor Osoti, Euniah Makori, Shehu Shagari, Chris Drakeley, Jonathan Cox, Teun Bousema

**Affiliations:** 1 Department of Infectious & Tropical Diseases, London School of Hygiene & Tropical Medicine, London, UK; 2 Department of Medical Microbiology, Radboud University Medical Center, Nijmegen, The Netherlands; 3 Centre for Global Health Research, Kenya Medical Research Institute and Centers of Disease Control and Prevention, Kisumu, Kenya; 4 Malaria Research Institute, Johns Hopkins Bloomberg School of Public Health, Baltimore, MD, USA

## Background

Mass screening and treatment campaigns have had limited success in curbing malaria transmission, possibly as a consequence of the high prevalence of subpatent infections that are missed using the currently available field based diagnostic tools. It has been shown that subpatent malaria infections are more likely to occur in households where patent infections are identified suggesting that it may be possible to employ a more focal approach to treatment campaigns using sentinel cases as markers for the presence of a subpatent reservoir. However, it is not known which definition of sentinel case to rely on for initiation of treatment would provide optimal coverage of the parasite reservoir.

## Materials and methods

In a low endemic and highly heterogeneous malaria transmission setting in the western Kenyan highlands we conducted a focal screening and treatment campaign as part of a larger cluster randomized trial. All consenting individuals under 15 years old and febrile adults residing within all households within the defined clusters were tested for malaria with a rapid diagnostic test (RDT). Temperature and blood spots on filter paper were collected from all household members (N = 2083), regardless of RDT result. The parasite reservoir was determined using nested polymerase chain reaction and gametocyte nucleic acid sequence-based amplification; multiple definitions of a sentinel case were assessed to determine which identified the largest proportion of parasitized individuals in the total population.

## Results

We observed significant household clustering of parasite carriage with a 23.5% (95% CI 21.6-25.3%) increased probability of being positive for malaria by PCR if another member of the same household was also parasitaemic. Sentinel populations of RDT-positive i) febrile individuals, ii) children <5 years of age, iii) children <15 years of age and iv) children <15 years of age and all febrile individuals have variable levels of coverage. However, even with the best approach (iv), only 36.0% to 80.0% of parasitized households corresponding to 46.6% to 90.1% of parasitized individuals were identified. Successful coverage was related to background parasitaemia and was improved considerably if compounds surrounding sentinel cases were included in the intervention.

## Conclusions

Despite strong clustering at the household level, targeting compounds with parasite positive sentinel cases does not achieve complete coverage of the parasitaemic reservoir. Even in the most optimistic scenario, 20% of households or 10% of the parasitaemic reservoir will go undetected when relying on conventional malaria diagnostics in sentinel populations.

